# Supplementary figures and images for: Extremes of age are associated with differences in the expression of selected pattern recognition receptor genes and ACE2, the receptor for SARS-CoV-2: implications for the epidemiology of COVID-19 disease
Source: BMC Med Genomics. 2021 May 24;14:138. doi: 10.1186/s12920-021-00970-7 (PMC8142073; doi:10.1186/s12920-021-00970-7)

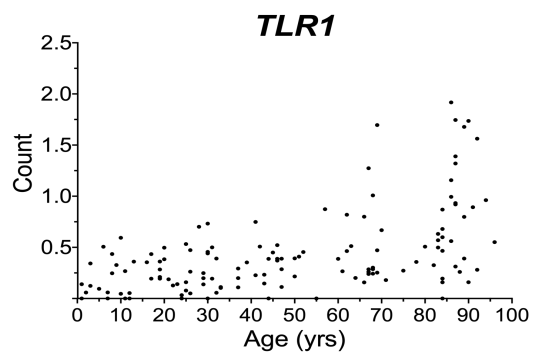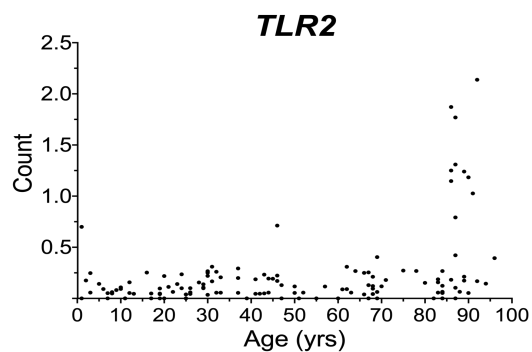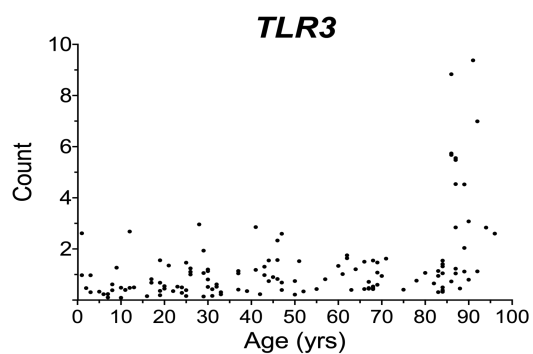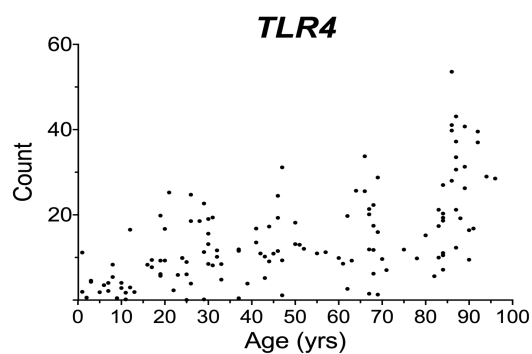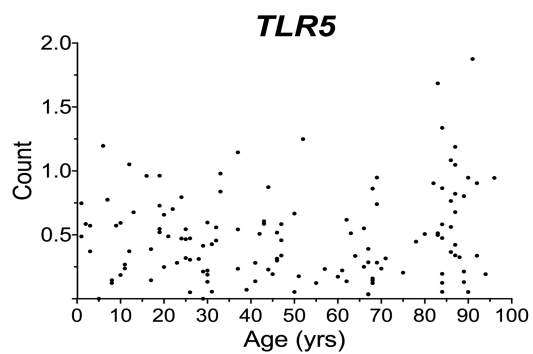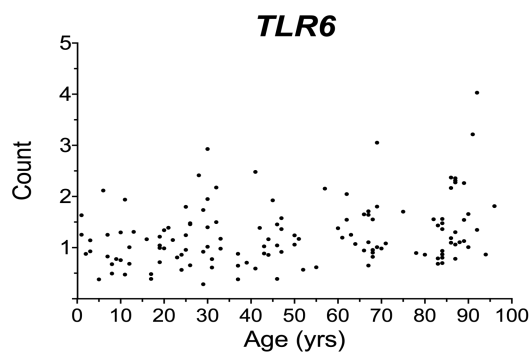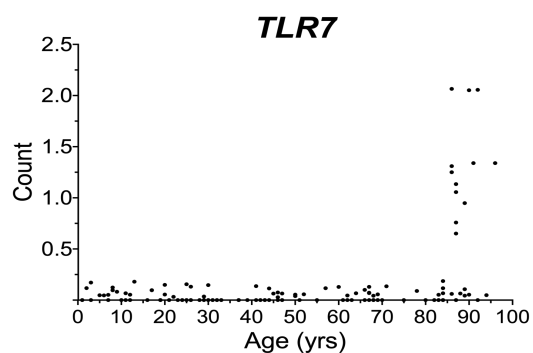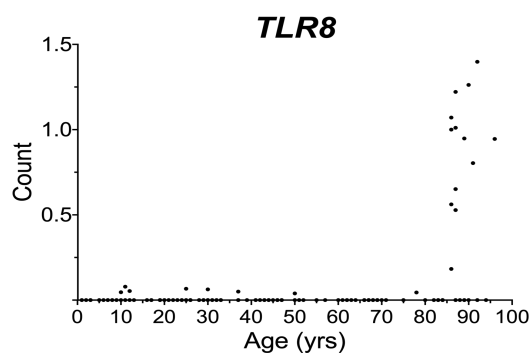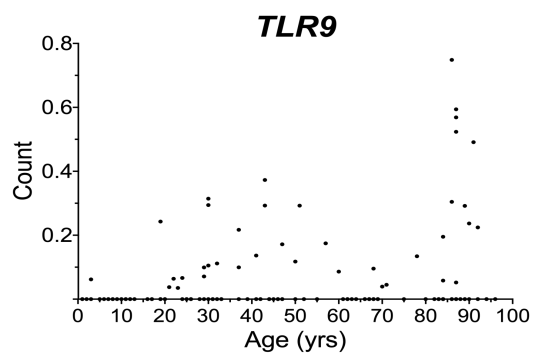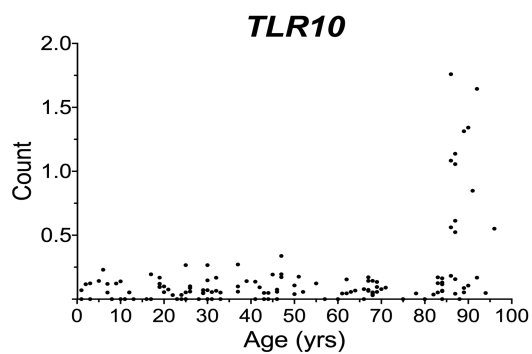

Supplement: Supplementary file 4 — Additional file 4. Supplementary Figure 1. Normalized gene counts for the ten Toll-like receptors expressed as a function of age [file 12920_2021_970_MOESM4_ESM.pdf]
